# The effects of primary care monitoring strategies on COVID-19 related hospitalisation and mortality: a retrospective electronic medical records review in a northern Italian province, the MAGMA study

**DOI:** 10.1080/13814788.2023.2186395

**Published:** 2023-04-20

**Authors:** Alice Serafini, Lucia Palandri, Peter Konstantin Kurotschka, Chiara Giansante, Maria Rita Sabattini, Martina Alfina Lavenia, Marina Scarpa, Davide Fornaciari, Matteo Morandi, Francesco Bellelli, Maria Stella Padula, Elena Righi, Giulia Ugolini, Silvia Riccomi, on behalf of the MAGMA Study Group

**Affiliations:** a Department of Primary Care, Local Health Authority of Modena, Modena, Italy; b Department of Biomedical, Metabolic and Neural Sciences, University of Modena and Reggio Emilia, Modena, Italy; c Department of General Practice, University Hospital Wuerzburg, Wuerzburg, Germany; d Department of Public Health, Local Health Authority of Bologna, Bologna, Italy; e Local Health Authority of Romagna, Ravenna, Italy

**Keywords:** COVID-19, general practice, hospitalisation, telemedicine, drug therapy

## Abstract

**Background:**

Most symptomatic SARS-CoV-2 infections produce mild to moderate symptoms. Although most patients are managed in the outpatient setting, little is known about the effect of general practitioners’ (GP) management strategies on the outcomes of COVID-19 outpatients in Italy.

**Objectives:**

Describe the management of Italian GPs of SARS-CoV-2 infected adult patients and explore whether GP active care and monitoring are associated with reducing hospitalisation and death.

**Methods:**

Retrospective observational study of SARS-CoV-2 infected adult outpatients managed by GPs in Modena (Italy) from March 2020 to April 2021. Information on management and monitoring strategies, patients’ socio-demographic characteristics, comorbidities, and outcomes (hospitalisation and death due to COVID-19) were retrieved through an electronic medical record review and analysed descriptively and through multiple logistic regression.

**Results:**

Out of the 5340 patients from 46 GPs included in the study, 3014 (56%) received remote monitoring, and 840 (16%) had at least one home visit. More than 85% of severe or critical patients were actively monitored (73% daily) and 52% were visited at home. Changes over time in patients’ therapeutic management were observed in concordance with the guidelines’ release. Active daily remote monitoring and home visits were strongly associated with reduced hospitalisation rate (OR 0.52, 95% CI 0.33–0.80 and OR 0.50, 95% CI 0.33–0.78 respectively).

**Conclusion:**

GPs effectively managed an increasing number of outpatients during the first waves of the pandemic. Active monitoring and home visits were associated with reduced hospitalisation in COVID-19 outpatients.

KEY MESSAGES

- This practice-based research network study from Northern Italy demonstrates the importance of primary care management of patients with COVID-19.
- GPs effectively treated a large - and increasing - number of patients outside the hospital.
- Active monitoring and home visits by GPs were associated with fewer hospital admissions for COVID-19.

## Introduction

Since February 2020, the Coronavirus disease 2019 (COVID-19) [1], challenged the Italian healthcare system heavily [2]. Patients with COVID-19 may have a wide range of clinical manifestations [3]. In most cases, however, patients develop a mild airway infection and therefore are commonly managed in the context of primary care (PC) by General Practitioners (GPs) [4]. In Italy, healthcare is provided through the National Health Service (NHS), in which GPs are responsible for providing free-of-charge care for most acute and chronic conditions.

Italian GPs faced several challenges and difficulties managing patients during the first wave of the COVID-19 pandemic [5]. The lack of personal protective equipment [6,7], and the absence of specific management guidelines impacted their capacity to provide care and led to widespread infections among these professionals, causing many fatalities [8,9].

Support for GPs was provided mainly by the GP-Out-of-Hour (OoH) service and by COVID-19 special units of continuity care (USCA-Unità Speciali di Continuità Assistenziale). These new working units were dedicated to home visits and initial assessment of infected patients [10].

To date, little is known about the management strategies used by Italian GPs for SARS-CoV-2 patients and their impact on COVID-19-related hospitalisations and mortality. First, this study aimed to describe the clinical characteristics of infected patients and the management strategies used by their GPs during the first and subsequent waves of the pandemic. Second, it aimed to explore the association between active monitoring by GPs and COVID-19-related hospitalisations and mortality in adult patients in the Province of Modena (Emilia-Romagna, Italy).

## Methods

### Study design

The MAGMA study is a retrospective cohort study of SARS-CoV-2 positive subjects based on the review of electronic medical records (EMRs) provided by 46 GPs of the Province of Modena (Italy). See the acknowledgement section for insights on the name MAGMA chosen for this study.

### Recruitment of GPs and selection of study subjects

Between March and April 2021, all GPs practising in the province of Modena (*n* = 462) were invited *via* e-mail to attend a study presentation webinar. During the webinar, GPs were asked to collaborate on the study. They were eligible if they recorded patient data using the software MILLEWIN^®^.

Participating GPs (*n* = 46) were provided with Structured Query Language (SQL) to perform EMR data extraction independently. SQL-based data extraction is routinely used by GPs in the local health service of Modena for audit purposes. Nevertheless, prior to the study, GPs received additional training on data wrangling to standardise activities and improve data quality. Data was checked for completeness and consistency by each GP, who manually added information on the severity of the disease and the monitoring strategies. We included in the analysis patients with a positive SARS-CoV-2 real-time polymerase chain reaction test performed between 01/03/2020 and 30/04/2021.

Residents in nursing homes were also included, as they are commonly managed by GPs. Patients who tested positive during a hospital stay were excluded.

### Variables

#### Exposure

The exposure of interest was the monitoring strategy adopted by each GP as follows. For remote (telephone) monitoring, 4 options were possible: (a) no monitoring, (b) passive monitoring (i.e. monitoring performed only upon the patient’s request); (c) irregular active monitoring (not daily); and (d) active daily or twice daily monitoring. There were also 4 options for home visits: (a) no home visits, (b) only USCA/OoH service visit; (c) only GP visit, or (d) both USCA/OoH service and GP visit. These options were then grouped into a summary variable to describe the overall GP monitoring strategy as follows: (a) none or low-level monitoring (patients with no monitoring, passive monitoring, or irregular active monitoring); (b) only active daily or twice daily remote monitoring; or (c) home visits (patients receiving at least one home visit, regardless remote monitoring).

#### Covariables

The patient’s socio-demographic characteristics, lifestyle and clinical features were collected: age, gender, number of comorbidities, severe obesity (BMI > 35), smoking habits, place of residence (home/nursing home), socio-economic deprivation (personal, social, or economic difficulties known by GP) (details of variable categories in Table 2), and any use of COVID-19 related therapies (Table 3). COVID-19 disease severity (asymptomatic, mild, moderate, severe or critical) was classified according to the United States National Institutes of Health Guidelines (NIH) (Tables 2 and 3) [11]. Finally, a binary variable was created to identify the infection period: first wave (from March to May 2020) or subsequent waves (June 2020 to April 2021).

#### Outcomes

The primary outcomes were COVID-19-related hospital admissions (yes/no) and COVID-19-related deaths (yes/no). Outcome-related variables were automatically registered in the patient’s electronic medical record. A complete description of the variables and their level definitions, the data sources and possible sources of bias are provided as Supplementary Material.

### Statistical analysis

Categorical variables were summarised by absolute and relative frequencies. Mean and Standard Deviation (SD) or median and interquartile range (IQR) were used to summarise continuous variables according to their distribution. Pearson’s chi-square test or Fisher’s exact test was used to compare categorical variables. *T*-student test or Mann–Whitney *U-*test was used to analyse numeric variables depending on their distribution. Multiple logistic regression models were built to explore the association of GPs´ monitoring strategies on hospitalisation or death due to COVID-19. The summary variable ‘GP monitoring strategy’ was used as the primary exposure variable in the model. To control for potential confounders and to evaluate their role, covariates were also included in the model. Associations are reported as adjusted odds ratios (aOR) and 95% confidence intervals (95% CI). SPSS Statistics®, version 27[12] and Excel®, version 16.0, were used to perform the analyses.

This study was reported according to Strengthening the Reporting of Observational Studies in Epidemiology (STROBE) guidelines [13].

## Results

### Characteristics of enrolled GPs

A total of 46 GPs (10% of all GPs in Modena) agreed to participate. They were younger, predominantly female, working in an urban setting and group practices. Enrolled GPs served a population of 64,763 people, with an average of 1408 (±399) patients each. Patients´ list sizes and age distributions were similar in participating and non-participating GPs (Table 1).

**Table 1.** Characteristics of General Practitioners (GPs) in the province of Modena (Italy) in 2020 stratified by GPs enrolled in the MAGMA study compared to non-participating GPs.

	GPs of Modena^a^	MAGMA Study GPs	Non-participating GPs^a^	p*
	462	46	416	
Mean age in years (SD)	58 (10)	51 (12)	n.a.	–
Gender				
Female	218 (47)	34 (74)	184 (44)	<.001
Male	244 (53)	12 (26)	232 (56)	
Practice’s organization				
Group practice	230 (50)	29 (63)	201 (48)	
Network practice^b^	174 (38)	17 (37)	157 (38)	.016
Solo practice	58 (12)	0 (0)	58 (14)	
Mean number of patients in the GP’s list (SD)				
Total number of patients	1323 (388)	1408 (399)	1314 (399)	.128
65–75 years	171 (61)	181 (50)	169 (62)	.271
>75 years	180 (71)	194 (59)	178 (72)	.135
Setting				
Urban	126 (27)	24 (52)	102 (25)	
Mixed	254 (55)	19 (41)	235 (56)	<.001
Rural	82 (18)	3 (7)	79 (19)	

Categorical variables are reported as number and column percentage, *n* (%), numeric variables as mean and standard deviation, mean (SD).

*Calculated using Pearson’s chi-square test or Fisher’s exact test when appropriate. n.a.: not available.

^a^Aggregated data on the GPs of Modena were provided by the local health authority. We calculated the values for non-participants by subtracting from the frequencies of the GPs of Modena, the frequencies of the MAGMA GPs. This was possible for all variables, except for the variable age.

^b^Network practice: GPs ensuring continuity of care among their patients but without sharing the same facility (group practice).

### Characteristics of patients

Data on 5340 infected patients were collected. Patients´ characteristics are reported in Table 2. GPs’ workload was significantly higher during the subsequent waves of COVID-19 pandemics, during which 5042 subjects (versus the 298 patients of the first wave) were managed, accounting for 94% of the entire sample. These patients were significantly younger, less socio-economic deprived, with fewer pre-existing comorbidities, and with a lower severity degree of the disease than those managed in the first pandemic wave.

**Table 2.** General and clinical characteristics stratified by the period of infection of patients with SARS-CoV-2 infection followed by general practitioners in Modena’s province (Italy) from March 2020 to April 2021.

		Total	First wave	Subsequent waves	p*
		5340	298	5042	
Age (years (±SD))		48 (±19)	56 (±18)	47 (±19)	<.001
Age (categories)	<18	282 (5)	1 (0)	281 (6)	<.001
	18–49	2594 (49)	107 (36)	2487 (49)	
	50–64	1423 (27)	102 (34)	1321 (26)	
	65–74	460 (9)	32 (11)	428 (8)	
	75–84	357 (7)	36 (12)	321 (6)	
	≥85	224 (4)	20 (7)	204 (4)	
Sex	Female	2784 (52)	163 (55)	2621 (52)	.371
	Male	2556 (48)	135 (45)	2421 (48)	
Smoking status	Active smoker	265 (5)	13 (4)	252 (5)	.361
	No smoker	5015 (95)	284 (95)	4731 (94)	
	Missing data	60 (1)	1 (0)	59 (1)	
Number of comorbidities	0	3661 (69)	177 (59)	3484 (69)	<.001
	1	1045 (20)	58 (20)	987 (20)	
	2	390 (7)	33 (11)	357 (7)	
	3	157 (3)	18 (6)	139 (3)	
	≥4	87 (2)	12 (4)	75 (2)	
Socio-economic deprivation^a^		280 (5)	25 (8)	255 (5)	.022
Residence	Nursing home	115 (2)	2 (1)	113 (2)	.095
	Home	5225 (98)	296 (99)	4929 (98)	
Severe obesity (BMI > 35)	Yes	244 (5)	18 (6)	226 (5)	.044
	No	4899 (95)	276 (94)	4623 (95)	
	Missing	197 (4)	4 (1)	193 (4)	
NIH COVID-19 stage	Asymptomatic	919 (17)	18 (6)	901 (18)	<.001
	Mild disease	2964 (56)	108 (36)	2856 (57)	
	Moderate disease	823 (15)	77 (26)	746 (15)	
	Severe disease	389 (7)	45 (15)	344 (7)	
	Critical disease	245 (5)	50 (17)	195 (4)	

First Wave: March 2020 – May 2020, Subsequent Waves: June 2020 – April 2021. Categorical variables are reported as number and column percentage, numeric variables as mean and standard deviation. NIH: US National Institute of Health.

^a^For definition refer to the covariable section in methods.

*Calculated using Pearson’s chi-square test or Fisher’s exact test when appropriate.

### Pharmacological strategies

Overall, 1413 (27%) patients received no pharmacological treatment. Significant prescription changes in in the subsequent waves over the first one were observed (Table 3). Antibiotics, corticosteroids, low molecular weight heparin (LMWH) and oxygen were prescribed more frequently in patients with pneumonia (NIH stage 2 or above), with significantly increased use during the subsequent waves. Anti-inflammatory drugs (NSAIDs) were more commonly prescribed in NIH stages 1 and 2 and their use increased in the successive waves while paracetamol showed a decreasing trend.

**Table 3.** General practitioners’ pharmacological strategies stratified by COVID-19 disease severity and by period of infection of patients with SARS-CoV-2 infection followed by general practitioners in Modena’s province (Italy) from March 2020 to April 2021.

Pharmacological treatment													
				Antibiotics		Antibiotics							
		*n*	None	Any^a^	Beta-lactam	Macrolide	Quinolones	LWMH	Oxygen	Steroid	NSAID	Paracetamol	HCQ
Overall	Total	5340	1413 (27)	1492 (28)	867 (16)	806 (15)	91 (2)	870 (16)	202 (4)	682 (13)	1566 (29)	2509 (47)	46 (1)
	First Wave	298	60 (20)	121 (41)	64 (1)	74 (25)	12 (4)	45 (15)	13 (4)	36 (12)	64 (22)	178 (60)	34 (11)
	Sub. Waves	5042	1353 (27)	1371 (27)	803 (15)	732 (15)	79 (2)	825 (16)	189 (4)	646 (13)	1502 (30)	2331 (46)	12 (0)
	p*		.011	<.001	.015	<.001	<.001	.628	.533	.789	.002	<.001	<.001
NIH stage 0	First Wave	18	12 (67)	3 (17)	2 (0)	1 (6)	1 (6)	1 (6)	0 (0)	0 (0)	0 (0)	2 (11)	0 (0)
Asymptomatic	Sub. Waves	901	657 (73)	67 (7)	44 (5)	28 (3)	4 (0)	22 (2)	0 (0)	11 (1)	82 (9)	110 (12)	1 (0)
	p*		.594	.152	.226	.442	.094	.369	–	1	.395	1	1
NIH stage 1	First Wave	108	28 (26)	29 (27)	17 (1)	18 (17)	2 (2)	5 (5)	0 (0)	3 (3)	28 (26)	62 (57)	4 (4)
Mild disease	Sub. Waves	2856	634 (22)	532 (19)	265 (9)	301 (11)	25 (1)	164 (6)	1 (0)	128 (5)	981 (34)	1469 (51)	5 (0)
	p*		.348	.044	.030	.056	.258	.832	1	.630	.078	.240	<.001
NIH stage 2	First Wave	77	8 (10)	47 (61)	21 (3)	32 (42)	2 (3)	13 (17)	0 (0)	9 (12)	16 (21)	54 (70)	20 (26)
Moderate disease	Sub. Waves	746	28 (4)	446 (60)	274 (33)	232 (31)	26 (4)	316 (42)	14 (2)	242 (32)	309 (41)	448 (60)	4 (1)
	p*		.014	.903	.106	.072	1	<.001	.633	<.001	<.001	.087	<.001
NIH stage 3	First Wave	45	2 (4)	28 (62)	16 (4)	16 (36)	3 (7)	18 (40)	7 (16)	13 (29)	13 (29)	26 (58)	7 (16)
Severe disease	Sub. Waves	344	16 (5)	221 (64)	146 (38)	121 (35)	14 (4)	226 (66)	105 (31)	180 (52)	97 (28)	202 (59)	2 (1)
	p*		1	.869	.424	1	.430	.002	.037	.004	1	1	.008
NIH stage 4	First Wave	50	10 (20)	14 (28)	8 (3)	7 (14)	4 (8)	8 (16)	6 (12)	11 (22)	7 (14)	34 (68)	3 (6)
Critical disease	Sub. Waves	195	18 (9)	105 (54)	74 (30)	50 (26)	10 (5)	97 (50)	69 (35)	85 (44)	33 (17)	102 (52)	0 (0)
	p*		.010	<.001	.004	.093	.493	<.001	<.001	.006	.830	.056	<.001

LWMH: low-weight-molecular-heparin; NSAID: non-steroid anti-inflammatory drugs; HCQ: Hydroxychloroquine. First Wave: March 2020 – May 2020, Subsequent (Sub.) Waves: June 2020 – April 2021), NIH: US National Institute of Health. Categorical variables are reported as number and percentage on *n*.

*Calculated using Pearson’s chi-square test or Fisher’s exact test when appropriate.

^a^Number of patients that received antibiotics, single or in combination.

### Monitoring strategies

Overall, 3014 (56%) patients received remote monitoring, either irregularly (9%), daily (24%) or twice daily (6%) (Table 4). Remote monitoring significantly decreased in subsequent waves; however, a similar prevalence of active monitoring in more severe patients was observed during the first and next waves, with more than 80% of stage 3 or 4 patients actively contacted by their GP (Table 4).

**Table 4.** General practitioners’ monitoring strategies stratified by COVID-19 disease severity and by period of infection of patients with SARS-CoV-2 infection followed by general practitioners in Modena’s province (Italy) from March 2020 to April 2021.

Monitoring strategies														
			Remote monitoring					Home visits				GP monitoring		
		*n*	None	PM	AM, irregular	AM, daily	AM, twice daily	None	USCA or OoH	GP	USCA + GPs	No low-level	AM, daily	Home visits
Overall	Total	5340	569 (11)	1757 (33)	1439 (27)	1275 (24)	300 (6)	4500 (84)	382 (7)	383 (7)	75 (1)	3470 (65)	1030 (19)	840 (16)
	First Wave	298	15 (5)	58 (20)	86 (29)	108 (36)	31 (10)	220 (74)	22 (7)	50 (17)	6 (2)	128 (43)	92 (31)	78 (26)
	Sub. Waves	5042	554 (11)	1699 (34)	1353 (27)	1167 (23)	269 (5)	4280 (85)	360 (7)	333 (7)	69 (1)	3342 (66)	938 (19)	762 (15)
	p*		<0.001					<.001				<.001		
NIH stage 0	First Wave	18	8 (44)	6 (33)	3 (17)	1 (6)	0 (0)	18 (100)	0 (0)	0 (0)	0 (0)	17 (94)	1 (6)	0 (0)
Asymptomatic	Sub. Waves	901	375 (42)	379 (42)	87 (10)	45 (5)	15 (2)	870 (97)	7 (1)	23 (3)	1 (0)	820 (91)	50 (6)	31 (3)
	p*		.818					.887				.725		
NIH stage 1	First Wave	108	3 (3)	29 (27)	44 (41)	31 (29)	1 (1)	95 (88)	8 (7)	4 (4)	1 (1)	69 (64)	26 (24)	13 (12)
Mild disease	Sub. Waves	2856	136 (5)	1144 (40)	1015 (36)	528 (19)	33 (1)	2671 (94)	59 (2)	116 (4)	10 (0)	2195 (77)	476 (17)	185 (7)
	p*		.017					.002				.005		
NIH stage 2	First Wave	77	0 (0)	14 (18)	24 (31)	32 (42)	7 (9)	56 (73)	7 (9)	10 (13)	4 (5)	27 (35)	29 (38)	21 (27)
Moderate disease	Sub. Waves	746	8 (1)	130 (17)	189 (25)	337 (45)	82 (11)	485 (65)	147 (20)	98 (13)	16 (2)	234 (31)	251 (34)	261 (35)
	p*		.698					.057				.398		
NIH stage 3	First Wave	45	0 (0)	4 (9)	9 (20)	20 (44)	12 (27)	28 (62)	5 (11)	12 (27)	0 (0)	8 (18)	20 (44)	17 (38)
Severe disease	Sub. Waves	344	14 (4)	29 (8)	43 (13)	173 (50)	85 (25)	170 (49)	89 (26)	60 (17)	25 (7)	54 (16)	116 (34)	174 (51)
	p*		.427					.019				.253		
NIH stage 4	First Wave	50	4 (8)	5 (10)	6 (12)	24 (48)	11 (22)	23 (46)	2 (4)	24 (48)	1 (2)	7 (14)	16 (32)	27 (54)
Critical disease	Sub. Waves	195	21 (11)	17 (9)	19 (10)	84 (43)	54 (28)	84 (43)	58 (30)	36 (19)	17 (9)	39 (20)	45 (23)	111 (57)
	p*		.866					<.001				.351		

PM: passive monitoring; AM: active monitoring; USCA: special units of continuity care; OoH: general practitioner Out of Hour Service; GP: general practitioner; First Wave: March 2020 – May 2020; Subsequent (Sub.) Waves: June 2020 – April 2021; NIH: US National Institute of Health. Categorical variables are reported as number and percentage on *n*.

*Calculated using Pearson’s chi-square test or Fisher’s exact test when appropriate.

**Figure 1.**
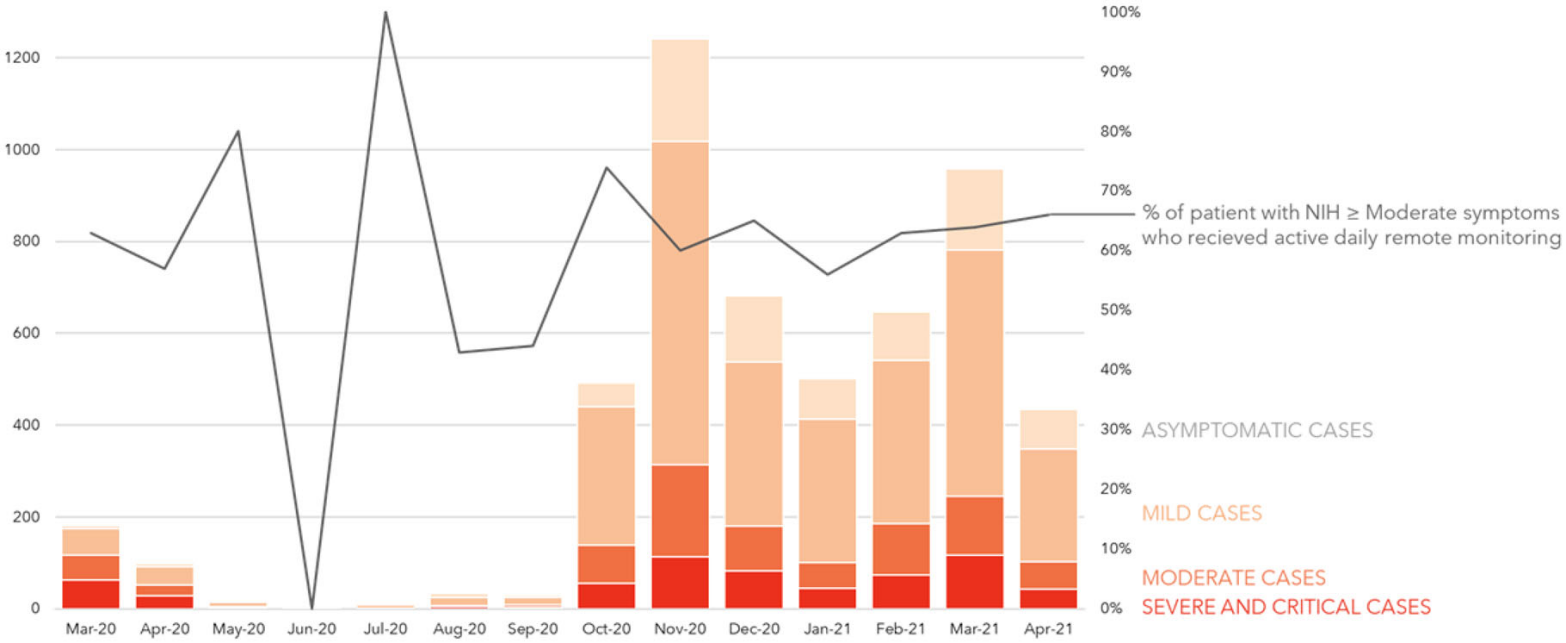
Absolute number of SARS-CoV-2 infected patients per month stratified by disease severity (coloured bars) managed by general practitioners (GP) in Modena’s province (Italy) from March 2020 to April 2021 in relation to the percentage of patients with pneumonia (NIH ≥ Moderate illness) who received GP’s active daily remote monitoring (dark line). NIH: US National Institute of Health.

Figure 1 shows a stable monthly percentage of active daily remote monitoring of pneumonia patients (more than 55%, dark line) over time, even with the increasing number of SARS-CoV-2 cases and consequent GP workload during the subsequent waves (coloured bars).

Overall, 840 patients (16%) were visited at home. Their proportion significantly increased with the increase of disease severity and in the subsequent pandemic waves (Table 4).

A similar trend was observed for the variable summarising the overall GP monitoring strategies.

### Hospitalisation and death for COVID-19

Overall, 521 patients (10%) were hospitalised (Table 5), with a significant reduction between the first and following waves (respectively 27% and 9%; *p* < .001). In the subsequent waves, the hospitalisation rate was lower regardless of the disease stage, as reported in Table 5.

**Table 5.** COVID-19 related outcomes (hospitalisation and death) stratified by COVID-19 disease severity and by period of infection of patients with SARS-CoV-2 infection followed by general practitioners in Modena’s province (Italy) from March 2020 to April 2021.

				
Outcomes		*N*	Hospitalisation (%)	Deaths (%)
Overall	Total	5340	521 (10)	114 (2)
	First Wave	298	80 (27)	27 (9)
	Sub. Waves	5042	441 (9)	87 (2)
	p*		<.001	<.001
NIH stage 0	First Wave	18	0 (0)	0 (0)
Asymptomatic	Sub. Waves	901	1 (0)	0 (0)
	p*		1	1
NIH stage 1	First Wave	108	0 (0)	0 (0)
Mild disease	Sub. Waves	2856	3 (0)	0 (0)
	p*		1	1
NIH stage 2	First Wave	77	2 (3)	0 (0)
Moderate disease	Sub. Waves	746	52 (7)	1 (0)
	p*		.222	1
NIH stage 3	First Wave	45	30 (67)	0 (0)
Severe disease	Sub. Waves	344	215 (63)	4 (1)
	p*		.626	.152
NIH stage 4	First Wave	50	48 (96)	27 (54)
Critical disease	Sub. Waves	195	170 (87)	82 (42)
	p*		.081	<.001

First Wave: March 2020 – May 2020; Subsequent (Sub.) Waves: June 2020 – April 2021. Categorical variables are reported as number and percentage on *n*.

*Calculated using Pearson’s chi-square test or Fisher’s exact test when appropriate.

Most hospital admissions concerned patients with the severe or critical disease (463, 89%), over 70 years (54%) or with at least one comorbidity (68%).

Overall, we observed 114 deaths (2%) due to COVID-19. Deaths were more frequent during the first wave than in the subsequent ones (respectively 9% vs 2%; *p* < .001) and occurred mainly in stage 4 (109 deaths, 96%). However, the mortality rate in critically ill patients decreased significantly in the subsequent waves compared to the first wave (Table 5).

Deaths occurred more frequently in older patients (mean ± SD: 82 ± 10 years), in those with 2 or more comorbidities (60%) and in males (56%). GPs managed twenty-seven deaths (24%) in an outpatient setting: 12 occurred at home upon the patient’s or caregiver’s request and 15 in nursing homes.

### GP monitoring strategies and hospitalisation and death due to COVID-19

As shown in Table 6, daily remote monitored or home-visited patients were half less likely to be hospitalised compared with non-monitored patients (aOR:0.52, and aOR: 0.50). A higher probability of hospitalisation was observed in older age, socio-economic deprivation, in patients with two or more comorbidities, and increasing COVID-19 illness severity. Living in a nursing home was associated with a significant reduction in the probability of hospitalisation. Mortality was not influenced by active daily monitoring or home visits. Death due to COVID-19 was associated with older age, infection in the first pandemic wave, living in a nursing home, having one or more comorbidities and increasing COVID-19 illness severity.

**Table 6.** Multiple Logistic Regression models of factors that influence the probability of hospitalisation and death for COVID-19 in patients with SARS-CoV-2 infection followed by general practitioners in Modena’s province (Italy) from March 2020 to April 2021.

		Hospitalisation for COVID-19		Death for COVID-19	
Variables		aOR (IC 95%)	*p*	aOR (IC 95%)	*p*
GP monitoring	No or low-level	Ref.		Ref.	
	Active daily	0.52 (0.33–0.80)	.003	2.15 (0.83–5.56)	.114
	Home visits	0.50 (0.33–0.78)	.002	0.85 (0.38–1.94)	.703
Sex	M	1.30 (0.95–1.80)	.103	1.16 (0.63–2.13)	.641
	F	Ref.		Ref.	
Age		1.02 (1.01–1.03)	.003	1.09 (1.05–1.12)	<.001
Obesity (BMI > 35)	Yes	1.11 (0.61–2.03)	.740	0.90 (0.28–2.84)	.851
	No	Ref.		Ref.	
Socio-economic deprivation	Yes	3.80 (2.24–6.43)	<.001	0.79 (0.33–1.87)	.590
	No	Ref.		Ref.	
COVID waves	First Wave	1.02 (0.60–1.72)	.946	2.11 (1.02–4.38)	.044
	Subsequent Waves	Ref.		Ref.	
Nursing home	Yes	0.12 (0.05–0.25)	<.001	18.83 (5.01–70.82)	<.001
	No	Ref.		Ref.	
Comorbidities	No	Ref.		Ref.	
	1	1.22 (0.82–1.82)	.324	3.10 (1.26–7.63)	.014
	2 or more	1.66 (1.04–2.65)	.034	2.60 (1.11–6.08)	.028
COVID-19 NIH stages	From asymptomatic to critical illness	19.41 (14.98–25.16)	<.001	132.24 (37.31–468.77)	<.001

## Discussion

### Main findings

This is the first study to describe the management strategies of GPs in a northern province of Italy in caring for SARS-CoV-2 infected patients. More than 85% of patients with the severe or critical disease were remotely monitored, 73% daily, and 52% received a home evaluation. In the subsequent waves, despite the exponential increase of COVID-19 cases to be managed, GPs increased the percentage of patients they could monitor *via* telephone or at home. In the following waves of the pandemic, the use of LMWH and oxygen increased in more severely ill patients, whereas the use of NSAIDs increased in the case of milder disease. As to the effects of GPs´ monitoring strategies, our analysis suggests that patients who get actively monitored or are home-visited are half less likely to be hospitalised than non-monitored patients.

### Strengths and limitations

Our study has several strengths. First, we included a large sample of patients obtained thanks to the broad collaboration of local GPs [14]. Second, our data source, the GPs EMR is widely considered a valuable source of information to conduct clinical research also considering the experience of GPs with this method of data extraction in Modena [15]. Third, this is one of the few studies in Italy describing how GPs responded to the current pandemic.

The main limitation is the retrospective nature of our study, not allowing us to establish causality. Second, we had no information on the timing of therapy or GP monitoring. This limited insights into patients’ management and disease pathways. Third, GPs participated voluntarily, so that, selection bias may affect generalisability. The differences observed between MAGMA GPs and those who did not may affect our results’ generalisability in the Province of Modena. Unfortunately, the lack of data on GP’s characteristics in Italy does not allow us to compare our GP sample nationally. Nevertheless, the characteristics of SARS-CoV-2 infected patients in terms of age, sex and clinical features in the first and subsequent waves reflect national data [16], so we believe that our findings’ significance is still valid.

### Interpretation and perspectives

During the first wave shortage of diagnostic tests [17], reserved for severe cases produced a selection of the infected population with different characteristics (older patients, with more comorbidities, with a higher hospitalisation and mortality rate) compared to subsequent waves [17,18]. Hospitalisation rate and deaths, as well, appear similar to those observed in another Italian study [19].

Further, therapeutic and monitoring strategies vary significantly between the first and next waves, consistently with the improved knowledge on the management of COVID-19 and the publication of guidelines from the Ministry of Health and the Italian regulatory agency (AIFA) [20,21]. Compared with the study conducted by Crisafulli et al. in southern Italy, our data showed an overall lower use of antibiotics (28% vs 52%), steroids (13% vs 36%) and oxygen (4% vs 7%) and a comparable use of heparin (16%) [19]. Also previous studies showed that GPs in northern Italy tend to prescribe less medications than in the south of the country, probably due to physicians’ attitudes [22–25]. In Italy, primary care was delivered in part by the GPs, and in part by the OoH/USCA service [26]. On the one hand this may limit the comparability of GPs’ management strategies we described herewith those other countries. On the other hand it represents an opportunity to learn from this different approach [27]. In any case, data on the clinical pathway of COVID-19 patients in primary care is lacking, both at national and international levels, making it difficult to quantify the contribution of primary care to the pandemic response and compare different strategies. This calls for further and more extensive studies of how GPs responded to the pandemic and what we can learn from this [28].

### Implications

Our study shows the benefits of proactive and integrated territorial management (both for remote monitoring and for home visits) on COVID-19 hospitalisation and the results are consistent with current literature [29]. Russo et al. showed a 70% decrease in hospitalisation in elderly patients from Northern Italy who were actively contacted by GPs during the infection compared non-contacted patients [30]. Effects on mortality rate do not appear so clear. Several factors, often unrelated to GPs’ monitoring, are potentially associated with patients’ death. The absence of explicit evidence of an impact on health outcomes, as mortality of remote patient monitoring, was described by a recent systematic review [29].

Potential protective effects of the different remote monitoring strategies, in place of or in addition to the in-person visits, need to be further investigated to draw guidelines to effectively relieve hospital pressure and costs.

GPs ability to adjust quickly in the face of a new pandemic may prove effective in helping the management of this and future epidemics. In these contexts, it is warranted that networks of primary care physicians and independent primary care practice-based research are strengthened, as they could promptly provide the much-needed evidence to implement rational patient management policies.

## Conclusion

Our findings described how GP responded to the first waves of the pandemic and suggest that active monitoring of COVID-19 outpatients performed by primary care physicians may reduce hospitalisations substantially.

## Supplementary Material

Supplemental Material

## Data Availability

The article’s data will be shared after a reasonable request to the corresponding author.
