# Supplementary material for: The effects of primary care monitoring strategies on COVID-19 related hospitalisation and mortality: a retrospective electronic medical records review in a northern Italian province, the MAGMA study
Source: Eur J Gen Pract. 2023 Apr 20;29(2):2186395. doi: 10.1080/13814788.2023.2186395 (PMC10249457; doi:10.1080/13814788.2023.2186395)
Supplement: Supplemental Material [file IGEN_A_2186395_SM6086.docx]

## The effects of primary care monitoring strategies on COVID-19 related hospitalisation and mortality: a retrospective electronic medical records review in a northern Italian Province, the MAGMA Study.

## Supplementary Material

## Source of data & variables

EMR: Electronic Medical Record, MRE: Medical Researcher Evaluation, MRI: Medical Researcher Integration, BMI: Body Mass Index; COVID-19: Coronavirus Disease 2019; NIH: National Institutes of Health; NSAIDs: Non-Steroidal Anti-Inflammatory Drugs; USCA: *Unità Speciali per la Continuità Assistenziale* (continuity of care special units); OoH: general practitioner Out of Hour Service, GP: general practitioner, HIV: Human Immunodeficiency Virus. Data about all GPs from the province of Modena were provided by the authors FB and MM from the Department of Primary Care of the Local Health Authority of Modena.

| General Characteristics and GP management strategies | | | |
| --- | --- | --- | --- |
| **Variables** | **Description of the variable** | **Data source** | **Systematic error** |
| Medical Researcher ID | Numeric, Identified through the Regional Code later encrypted | EMR | None |
| Patient ID | Anonymized alphanumeric code | EMR | None |
| Age | Numeric | EMR | None |
| Sex | Male, Female | EMR | None |
| Comorbidities | For extracted comorbidities see table below | EMR | See below |
| Smoking status | Active smoker or no smoker | EMR | Missing data possible,  Variable prone to response bias |
| Socio-economic deprivation | Dichotomous (y/n) Difficulties in the disease’ management due to personal, social, economic, or technological problems | MRE | Medical judgment  Reduced by: material-supported training to uniform the judgment of researchers |
| Severe Obesity | Dichotomous (y/n), defined as  yes if > 35 Kg/m2 or  no if < 35 Kg/m2 | EMR | Missing data possible |
| Residence | Patient who permanently lives in a Residential Care Facility or at home | EMR | None |
| NIH COVID-19 stage | Disease severity classification according to NIH guidelines^[[1]](#footnote-1)^:  0= Asymptomatic,  1= Mild disease,  2=Moderate disease,  3= Severe disease,  4= Critical disease. | MRE | Medical judgment  Reduced by: material-supported training to uniform the judgment of researchers |
| Remote medical monitoring | Defined as  0=no telemonitoring, 1=passive monitoring, wait for patient’s call;  2= irregular active remote monitoring;  3=regular active, daily monitoring;  4=regular active, bi-daily | MRE | Medical judgment  Reduced by: material-supported training to uniform the judgment of researchers |
| USCA visits | Dichotomous (y/n) Home visit from a doctor of *Unità Speciali per la Continuità Assistenziale* (continuity of care special units) | EMR | None |
| OoH visits | Dichotomous (y/n) | EMR | None |
| GP visits | Dichotomous (y/n) General Practitioner home visit | MRI | Medical judgment Reduced by: material-supported training to uniform the judgment of researchers |
| Hospitalization | Dichotomous (y/n) Hospital admission due to COVID-19 | EMR | None |
| COVID-19 related death | Dichotomous (y/n) | EMR | None |

## ATC Codes for Pharmacological treatment extracted from general practitioners’ EMR

Prescriptions were manually checked to ensure that they were related to the SARS-CoV-2 infection.

| **DRUG** | **ATC CODE** |
| --- | --- |
| Oxygen* | V03AN01 |
| Enoxaparin | B01AB05 |
| Prednisone | H02AB07 |
| Systemic Steroids | H02A |
| Beta-lactam antibacterials, penicillin | J01C |
| Other beta-lactam antibacterials | J01D |
| Macrolides | J01FA |
| Fluoroquinolones | J01MA |
| Warfarin | B01AA03 |
| [Direct factor Xa inhibitors](https://www.whocc.no/atc_ddd_index/?code=B01AF&showdescription=no) | B01AF |
| Dabigatran | B01AE07 |
| Hydroxychloroquine | P01BA02 |
| Paracetamol* | N02BE01 |
| Coxibs | M01AH |
| Metamizole Sodium | N02BB02 |
| Non-Steroidal Anti-Inflammatory Drugs (NSAIDs)* | M01A |
| Oxicams | M01AC |
| Formoterol* and budesonide* | R03AK07 |

** Underestimation of data possible in case of non-electronic prescription*

## Comorbidities extracted from general practitioners’ EMR

| **CONDITION** |  |
| --- | --- |
| Cerebrovascular disease | ICD 9 code 437.9/00 |
| Thalassemia | ICD 9 code 282.4/00 |
| Spherocytosis | ICD 9 code 282.0/09 |
| Pregnancy | ICD 9 code V22.2/06 |
| HIV infection | ICD 9 codes V08/85 , 042/00 |
| Diabetes | Textual research* |
| Dementia | Textual research* |
| Chronic Kidney Disease | Textual research* |
| Heart failure | Textual research* |
| Asthma or Chronic obstructive pulmonary disease | Textual research* |
| Vascular disease | Textual research* |
| Neoplasm (any) | Textual research* |
| Down syndrome | Textual research* |
| Immunodeficiency | Textual research* |
| Chronic Hepatitis | Textual research* |
| Organ transplantation | Textual research* |
| Dialysis | Textual research* |
| Myocardial infarction | Textual research* |

**Comorbidities extracted via textual research of keywords (ex: “diabetes”) in textual diagnosis of EMR.*

1. «COVID-19 Treatment Guidelines Panel. Coronavirus Disease 2019 (COVID-19) Treatment Guidelines. National Institutes of Health. Available at https://www.covid19treatmentguidelines.nih.gov/. Accessed 12/09/2021», s.d., https://files.covid19treatmentguidelines.nih.gov/guidelines/covid19treatmentguidelines.pdf. [↑](#footnote-ref-1)
